# Cytotoxicity of the Aqueous Extract and Organic Fractions from *Origanum majorana* on Human Breast Cell Line MDA-MB-231 and Human Colon Cell Line HT-29

**DOI:** 10.1155/2018/3297193

**Published:** 2018-08-23

**Authors:** Hanane Makrane, Mouhcine El Messaoudi, Ahmed Melhaoui, Mohammed El Mzibri, Laila Benbacer, Mohammed Aziz

**Affiliations:** ^1^Laboratory of Physiology, Genetic and Ethnopharmacology, Faculty of Sciences, Mohammed the First University, PB 717, 60000 Oujda, Morocco; ^2^Biology Unit and Medical Research CNESTEN, 10001 Rabat, Morocco; ^3^Laboratory of Organic Chemistry, Macromolecular and Natural Products, Faculty of Sciences, Mohammed the First University, PB 717, 60000 Oujda, Morocco

## Abstract

The toxicity of the aqueous extract of *Origanum majorana* was tested (5 and 10 g/kg) in albino mice. No symptoms of toxicity or mortality were observed. The mice survived being active and healthy during all 14 days of observation. In addition, the weight measurement of the left and right kidneys, heart, and liver shows no significant difference between the control, 5 g/kg, and 10 g/kg. All extracts (aqueous, petroleum ether, dichloromethane, ethyl acetate, methanolic, and depleted aqueous extracts) of *Origanum majorana* tested against both types of cancer cells showed a more pronounced cytotoxic effect against breast cell line MDA-MB-231 than colon cells line HT-29 cells. The most marked effect is that of the ethyl acetate extract with IC_50_ 30.90 ± 1.39 and 50.11 ± 1.44 (*µ*g/ml), respectively. HPLC analysis of extracts from *Origanum majorana* showed that this plant contained polyphenols and flavonoids, which may be responsible for the biological activities found.

## 1. Introduction

Worldwide, cancer is a great public health problem. It is the third leading cause of death in middle- and low-income countries [1]. During last decades, cancer therapy has shown a great evolution at both conceptual and methodological levels, using new developed drugs and high-efficient protocols. However, these therapies cause serious unwanted side effects, and most patients do not experience long-term remission [2]. Therefore, discovering new drugs with minimal toxicity is a wide scientific challenge.

A clear revival of interest to herbal medicine and development of novel phytochemical anticancer agents have gained significant recognition in the field of cancer therapy. In this field, medicinal plants represent one of the main sources of active compounds with antiproliferative and anticancerous activities [3]. In Morocco, the use of plants in the form of infusions or decoctions is a common practice among people of rural communities, and their use is increasing in urban populations. Among them, *Origanum majorana* (Lamiaceae) is an aromatic plant native to the Mediterranean region. In the Moroccan traditional medicine, this plant is widely used for the treatment of digestive disorders, flatulence, nausea, intestinal spasms, diarrhea, nervous disorders, migraines, insomnia, joint pain, cramps, body aches, inflammations of the oral mucous membrane, and dental and respiratory problems [4]. Several Scientific studies have confirmed some of these biological activities [5–9]. *Origanum majorana* is a plant widely used not only in culinary preparations but also for its medicinal properties as an antiseptic. Several research studies have shown that plants used in traditional medicine have a cytotoxic effect on cancerous lines and potentially have an antiproliferative effect. In addition, given its richness in phytoconstituents such as polyphenols and flavonoids, we have judged interesting to explore its cytotoxic effect to expand after other specific studies its use in traditional medicine.

The objective of the present study is to evaluate the oral acute toxicity of *Origanum majorana* collected in Morocco and its potential cytotoxic against human breast MDA-MB-231 and colon HT-29 cell lines. The choice of both lines is justified by the fact that breast and colon cancers are among the major cancers in Morocco, and therefore, we chose to work on human cancer lines derived from these two cancers.

## 2. Materials and Methods

### 2.1. Plant Material

The medicinal plant used in this study is *Origanum majorana* L. The aerial parts used of this plant were collected in January in the region of Errachidia, Morocco. It was identified by Professor Fennane Mohammed, from Scientific Institute in Rabat, Morocco, and registered under voucher number HUMPOM83 at the plant section of Herbarium, University Mohammed Premier, Oujda, Morocco.

### 2.2. Preparation of Extracts

The aqueous extract of *Origanum majorana* (AEOM) was prepared according to traditional use by the Moroccan population as an infusion. The yield of this extraction was 30.96%. Organic extraction with different solvents was also performed. The yields by extracting with petroleum ether, dichloromethane, ethyl acetate, methanol, and distilled water were 5.15%, 3.25%, 2.05%, 20.6%, and 7.1%, respectively.

### 2.3. Chemicals

Solvents used in the experiments were purchased from Sigma-Aldrich. Authentic standards of phenolic compounds were purchased from Sigma and Fluka. All chemicals were of analytical grade.

### 2.4. Preparation of Standard Solutions

Stock standard solutions were prepared by accurately weighing 10 mg of caffeic acid, chlorogenic acid, *p-*coumaric acid, *trans*-ferulic acid, cinnamic acid, quercetin, luteolin, apigenin, rutin, vanillic acid, syringic acid, *o*-coumaric acid, catechin, vanillin, *trans*-chalcone, 4-hydroxybenzoic acid, 2-(4-hydroxyphenyl)ethanol, and 3,4-dihydropyran reference standards into separate 2 ml volumetric flasks and dissolving in methanol. All samples were filtered through a 0.45 *μ*m membrane filter before injection.

### 2.5. HPLC Analysis of Phenolic Compounds

Analysis of phenolic compounds was carried out using an alliance apparatus, with separation module; Waters e2695 and detector PDA; and Waters 2998. The separation was carried out on 250 mm × 4.6 mm, 5 *µ*m C18 column at ambient temperature. The mobile phase consisted of ultrapure water/acetic acid (0.5%) (solvent A) and methanol (solvent B). The flow rate was kept at 1 ml/min. The gradient programme was as follows: 80% A/20% B 0–20 min, 0% A/100% B 20–25 min, 0% A/100% B 25–35 min, and 80% A/20% B 35–40 min. The injection volume was 20 *µ*l, and peaks were detected in scan mode between 210 and 500 nm. Peaks were identified by comparison of their relative retention times and UV spectra with those of authentic standards analysed in the same conditions.

### 2.6. Acute Toxicity Test

In this study, three lots of six albino mice (3♂ and 3♀ in each lot) were used to evaluate the acute toxicity of AEOM, according to the Lorke-written method with some modifications [10]. Mice were fasted for 18 h with free access to water before experience. The extract was dissolved in distilled water and administered via the oral route (5 and 10 g/kg). After treatment, mice are fed and followed by weight registration and observation of general signs of toxicity symptoms, behavior, and mortality for 14 days. At the end of the study, mice were sacrificed by cervical dislocation and organs such as right and left kidneys, liver, and heart were weighed. The appearance of the intestines and stomach was also observed [11]. All procedures concerning animals were carried out in an ethically proper way by following guidelines as set by the World Health Organization and conform to the European Community guiding principles in the care and use of animals (86/609/CEE, CE Off J no. L358, 18 December 1986).

### 2.7. Cell Lines and Cell Cultures

Cell lines used in this study included the human breast cell line MDA-MB-231 and human colon cell line HT-29. Cancerous cell lines were cultured in DMEM supplemented with 1% glutamine (Gibco), 10% fetal calf serum (Gibco), and 1% of mixture of streptomycin/penicillin. Cells were maintained at 37°C with 5% CO_2_ and 95% humidity. Confluent cells are trypsinized and then subcultured at lower numbers in new culture flasks.

### 2.8. *In Vitro* Cytotoxicity Assay

Cell viability was estimated on the basis of mitochondrial metabolic activity using WST1 (disodium mono{4-[3-(4-iodophenyl)-2-(4-nitrophenyl)-2H-tetrazol]-3-ium-5-yl] benzene-1.3-disulfonate}) reagent (Roche Applied Science), according to the manufacturer's protocol.

Exponentially growing MDA-MB-231 and HT-29 cells were seeded at a density of 8000 cells per well in 96-well plates. After 24 h, the culture medium was replaced by an experimental one with different concentrations of extract (initially dissolved in DMSO), ranging from 15.6 to 500 *μ*g/ml, in duplicate, and reincubated for 72 h. Following incubation, 100 *μ*l of the medium was aspirated, 10 *μ*l of WST1 reagent was added, and the plate was incubated for further 4 h. Cell viability was assessed by absorbance reading of each well at 450 nm using a Wallac Victor X3 multiplate reader. In these tests, the medium without extract and mitomycin C were used as negative and positive controls, respectively. Data are expressed as percentages of absorbance between treated and untreated (negative control) cells. Data are presented as means ± SD of assays performed in duplicate.

### 2.9. Statistical Analysis

Results are expressed as mean ± SEM. Statistical analysis was performed by Student's *t*-test using Microsoft Excel software. Significant differences are obtained when *p* value was below 0.05.

## 3. Results

### 3.1. High-Performance Liquid Chromatographic (HPLC) Separation and Determination of Main Phenolic Compounds in Aqueous and Organic Plant Extracts

**Figure 1:**
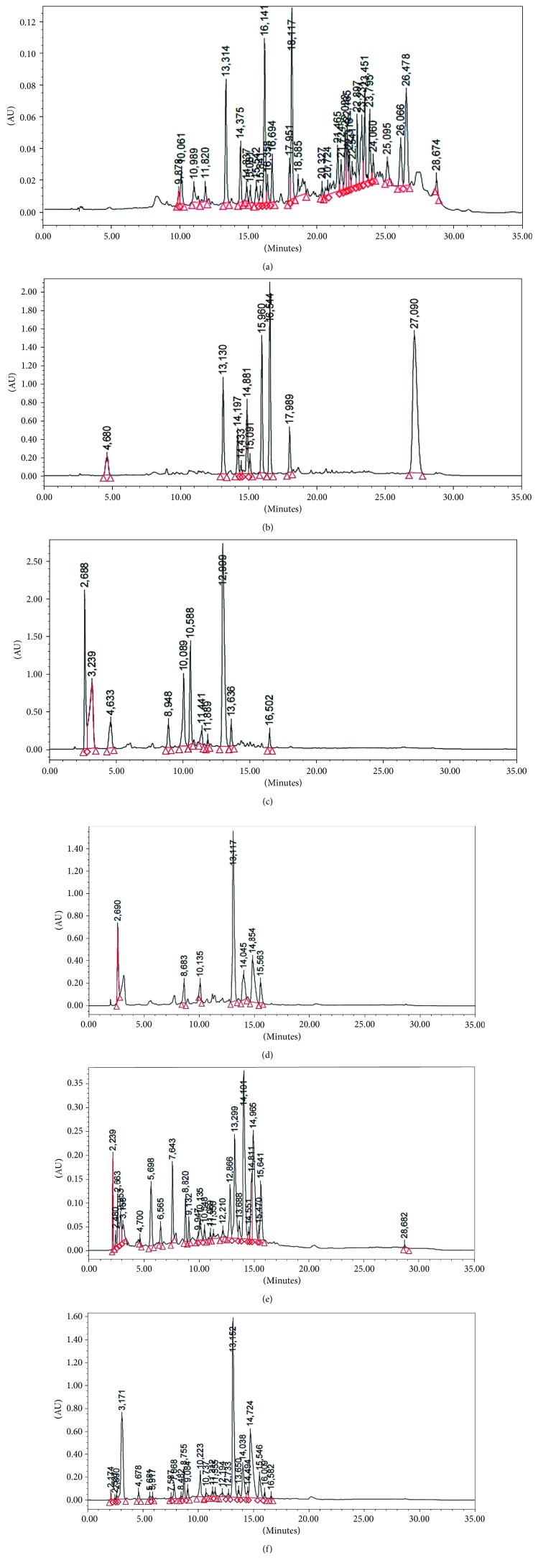
HPLC chromatogram of petroleum ether (a), dichloromethane (b), ethyl acetate (c), methanol (d), depleted aqueous (e), and aqueous (f) extracts from *Origanum majorana*.

Qualitative analysis for the identification of specific phenolic compounds was carried out via HPLC. According to the retention time of calibration standards (Table 1), aqueous and organic extracts of *Origanum majorana* presented the same chemical profile composed of 18 identified phenolic compounds present in some fractions and absent in the other. The chromatograms also show some other peaks apart from the 18 standards studied (Figure 1; Table 2). It should be noted that *trans*-ferulic acid was present in all fractions, the same for coumaric acid, but it was not present in the ethyl acetate fraction.

### 3.2. Acute Toxicity Test

**Figure 2:**
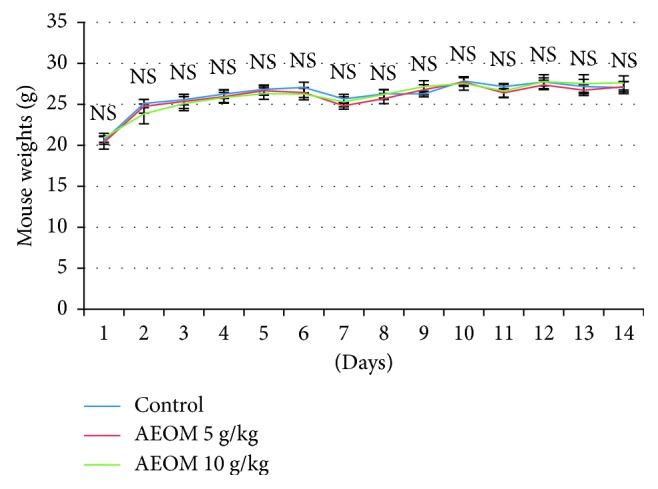
Effect of the aqueous extract of *Origanum majorana* on the weight of mice during the acute toxicity test. NS = not significant compared to control (distilled water) (mean ± SEM, *n*=6).

The AEOM showed no mortality with doses 5 and 10 g/kg body weight after 14 days of observation and no difference in the total weight of the mice daily measured between the control and the treated groups (Figure 2). Moreover, no modification in mice behaviors was registered, and no toxicity symptoms were reported during the study period.

**Figure 3:**
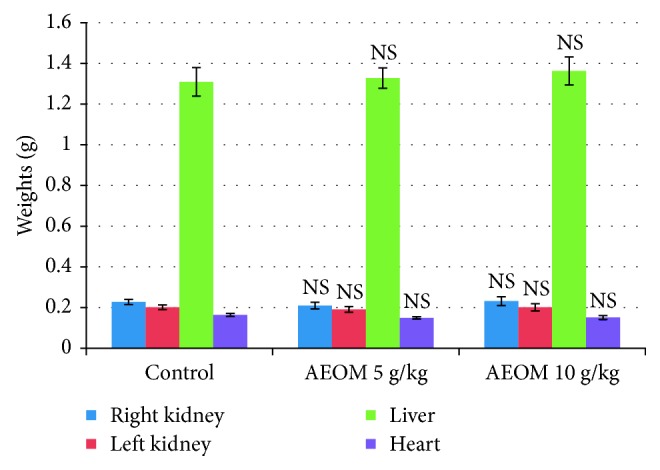
Effect of the aqueous extract of *Origanum majorana* on organs' weights of mice during the acute toxicity test. NS = not significant compared to control (distilled water) (mean ± SEM, *n*=6).

At the end of the toxicity test, mice were sacrificed by cervical dislocation; right and left kidneys, liver, and heart were recovered and weighed (Figure 3). Data analysis showed no significant difference between the control and treated groups. Visual evaluation of stomachs and intestines of mice showed normal appearance.

### 3.3. *In Vitro* Cytotoxicity Assay

Initially, the effect of the extracts on the morphology of cells can be visualized under phase-contrast inverted microscope, after incubation of cells with increasing concentrations of extracts [12]. Treatment of breast MDA-MB-231 and colon HT-29 human cancer cell lines for 72 h with increasing concentrations of extracts from *Origanum majorana* causes morphological changes (detached cells and loss of anchor properties) compared to untreated cells (cells attached to neighbors and preservation of anchor properties), revealing that both lines went through cell death with pronounced effect on MDA-MB-231 cells (data not shown).

The cytotoxic activity of the six *Origanum majorana* extracts against both human cancer cell lines was then investigated using a WST-1 viability assay. The 50% inhibitory concentrations (IC_50_) for each extract was determined.

**Figure 4:**
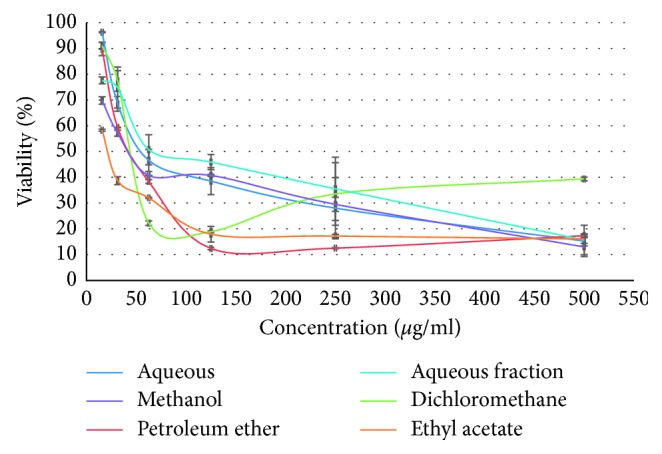
Effect of *Origanum majorana* extracts on cell proliferation of MDA-MB-231 cell line presented as percentage of cell viability and index, versus concentration of the extracts.

**Figure 5:**
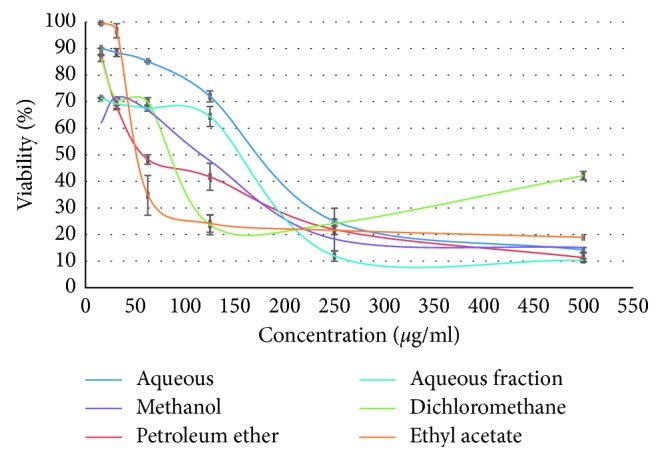
Effect of *Origanum majorana* extracts on cell proliferation of HT-29 cell line presented as percentage of cell viability and index, versus concentration of the extracts.

Using cell viability indices, the WST-1 test revealed that all extracts reduced cell viability in MDA-MB-231 (Figure 4) and HT-29 (Figure 5) cell lines in a dose-dependent manner after 72 h of treatment (studied range 15.6 to 500 *µ*g/ml), with a pronounced effect of ethyl acetate extract in both lines. The obtained IC_50_ for each extract in both MDA-MB-231 and HT-29 cell lines is summarized in Table 3.

Of particular interest, breast cancer cells MDA-MB-231 were more sensitive to the effect of *Origanum majorana* extracts than colon cancer cells HT-29. In MDA-MB-231 cell line, cell viability decreased drastically at higher concentrations of plant extracts with IC_50_ ranging from 30.90 to 87.09 *µ*g/ml. In HT-29 cells, the impact is less pronounced, with IC_50_ ranging from 50.11 to 158.48 *µ*g/ml. We also noted that the organic fractions of this plant are more active substances, than aqueous and depleted aqueous extracts.

For both assays, statistical analysis showed that the difference between cell viability obtained with tested extracts and negative control was significant (*p* ≤ 0.05).

## 4. Discussion

The toxicity of the AEOM (5 and 10 g/kg) was evaluated in albino mice. Obtained results clearly showed the absence of mortality and any symptom of toxicity. The mice survived being active and healthy during all 14 days of observation. In addition, the weight measurement of the left and right kidneys, heart, and liver showed no significant difference between the control and assays with 5 g/kg and 10 g/kg body weight. Therefore, the predicted LD_50_ of AEOM will be higher than 10 g/kg. The method of Dragstedt and Lang states that “Any animal that has survived at a given dose would have survived in any dose lower than this, if it had been administered to him [13],” and the classification of Loomis and Hayes suggests that a chemical with an LD_50_ between 5,000 and 15,000 mg/kg is considered to be nontoxic [14]. We can assume that AEOM and consequently *Origanum majorana* should be considered practically nontoxic in case of acute ingestion. These results are consolidated by the no difference in the weight of the different organs of the control and the treated groups. Taken together, these results are good indicators of the absence of toxicity of AEOM suggesting the safety of *Origanum majorana* for daily use by the Moroccan population mainly for flavoring tea.

*In vitro* cytotoxic tests showed that *Origanum majorana* extracts inhibit the proliferation of MDA-MB-231 and colon HT-29 human cancer cell lines in a dose-dependent manner, with pronounced effect on breast cancer cells MDA-MB-231.

Our results are in agreement with previous reported studies which highlighted the cytotoxic effect of *Origanum majorana* against the same lines. Research work of Al Tamimi showed that in the colon cancer cell line HT-29, *Origanum majorana* extracts potently inhibited tumor cell growths by inducing DNA damage, growth arrest, and activating apoptosis in the colon cancer cells [15]. In MDA-MB-231 cells, the effect of *Origanum majorana* extracts on the cell proliferation has been already discussed and highlighted the presence of various cell death mechanisms. Al Dhaheri et al. showed that treatment with noncytotoxic concentrations of *Origanum majorana* extracts inhibits migration and invasion of cells through inactivation of the NFKB pathways [16], while exposure of MDA-MB-231 cells to low concentration of ethanolic extracts (150 and 300 *µ*g/ml) from *Origanum majorana* triggers the accumulation of apoptotic-resistant population of cells arrested in mitosis and induces an over expression of survivin, an important therapeutic target against breast cancer [17]. On the other hand, treatment of MDA-MB-231 cells to higher concentration (400 and 600 *µ*g/ml) induces a down regulation of survivin and activation of caspase and PARP pathways [17].

Others studies have reported that other molecular mechanisms are affected by *Origanum majorana* extracts, including upregulation of E-cadherin, activation of NF-α, and decrease of the expression of metalloproteinases (MMP-2 and MMP-9), urokinase plasminogen activator receptor (uPAR), ICAM-1, and VEGF [18].

The anticancer potential of *Origanum majorana* has also been reported on other cancer cells such as fibrosarcoma cells (HT-1080) [19] and mammary adenocarcinoma cells (AMN-3 cell line) [20]. Interestingly, *Origanum majorana* extracts at concentration ranging from 1 to 2 mg/ml have a cytotoxic effect on human leukemic cell line jurkat and induces apoptosis with upregulation of p53 protein levels and down regulation of BcL2-alpha [21].

Other biological activities of *Origanum majorana* are also reported, including antimicrobial activity [8] and antioxidant activity associated with free radicals scavenging potential [7].

Lamiaceae species are known to contain a range of secondary metabolites, such as arbutin [22], tannins, flavonoids, sitosterol, phenolic glycosides, phenolic terpenoids [23], and numerous essential oil molecules [24]. HPLC analysis showed the presence of polyphenols and flavonoids in the organic fractions and the aqueous extract of *Origanum majorana* such as caffeic acid, chlorogenic acid, *p*-coumaric acid, *trans*-ferulic acid, cinnamic acid, quercetin, luteolin, apigenin, rutin, vanillic acid, syringic acid, *o*-coumaric acid, catechin, vanillin, *trans*-chalcone, 4-hydroxybenzoic acid, 2-(4-hydroxyphenyl)ethanol, and 3,4-dihydropyran. This phytochemical study was also reported by several studies [25–27]. The biological properties of *Origanum majorana* appear to be attributed to this chemical composition by the action of a single compound or by a synergistic or potentiation effect of all these compounds. Indeed, ferulic acid, *p*-coumaric acid, and rutin in previous studies showed anticancer effects [28–30].

## 5. Conclusions

In conclusion, this study is very informative: (i) aqueous extract of *Origanum majorana* is nontoxic in case of acute ingestion; (ii) methanolic, petroleum ether, ethyl acetate, dichloromethane, aqueous, and depleted aqueous extracts have a cytotoxic activity on MDA-MB-231 breast cancer and HT-29 colon cancer cell lines; (iii) the activity of *Origanum majorana* extracts is more pronounced in MDA-MB-231 breast cancer cell line; and (iv) *Origanum majorana* contains polyphenols and flavonoids that may be responsible for the biological activities found.

## Figures and Tables

**Table 1:** Retention time of 18 standard phenolic acids and flavonoids, and results of HPLC analysis of *Origanum majorana* extracts with different solvents.

Standards	RT (min)	Petroleum ether	Dichloromethane	Ethyl acetate	Methanol	Depleted aqueous	Aqueous
Chlorogenic acid	9.03	−	−	−	−	−	+
Catechin	9.13	−	−	−	−	+	+
3,4-Dihydropyran	9.50	+	−	−	−	+	−
2-(4-Hydroxy phenyl) ethanol	10.20	+	−	+	+	+	+
Vanillic acid	10.33	−	−	+	+	−	+
4-Hydroxybenzoic acid	10.42	−	−	+	−	−	+
Syringic acid	10.51	+	−	+	−	+	+
Caffeic acid	11.01	+	−	+	−	+	+
Vanillin	11.25	+	−	+	−	+	+
*p*-Coumaric acid	12.63	−	−	*−*	−	+	+
*trans*-Ferulic acid	13.25	+	+	+	+	+	+
Rutin	15.22	+	+	*−*	+	−	+
*o*-Coumaric acid	15.27	+	+	*−*	+	+	+
Luteolin	16.54	+	+	+	−	−	+
Cinnamic acid	16.62	+	+	+	−	−	+
Apigenin	17.36	−	+	−	−	−	−
Quercetin	17.82	+	+	−	−	−	−
*trans*-Chalcone	20.45	+	−	−	−	−	−

“+” sign indicates the presence of phenolic compounds; “−” sign indicates the absence of phenolic compounds.

**Table 2:** Molecular formula of the phenolic compounds of the aqueous and organic extracts of Moroccan marjoram.

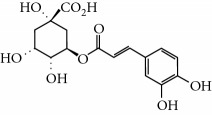 Chlorogenic acid	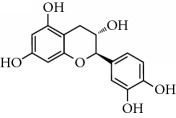 Catechin	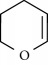 3,4-Dihydropyran
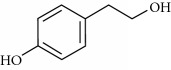 2-(4-Hydroxyphenyl) ethanol	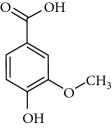 Vanilic acid	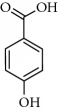 4-Hydroxybenzoic acid
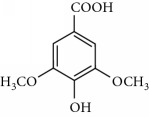 Syringic acid	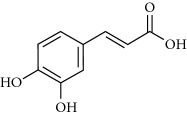 Caffeic acid	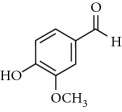 Vanillin
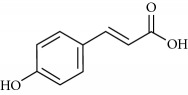 *p*-Coumaric acid	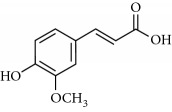 *trans*-Ferulic acid	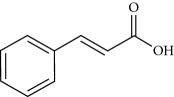 Cinnamic acid
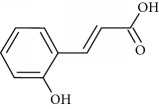 *o*-Coumaric acid	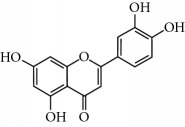 Luteolin	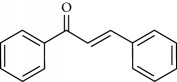 *trans*-Chalcone
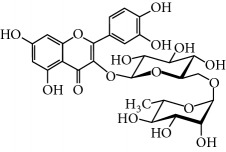 Ruttin	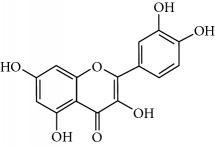 Quercetin	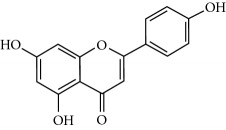 Apigenin

**Table 3:** IC_50_ values of *Origanum majorana* extracts in both MDA-MB-231 and HT-29 cell lines.

*Origanum majorana* extracts	IC_50_ values (*µ*g/ml)	
	MDA-MB-231	HT-29
Ethyl acetate	30.90 ± 1.39	50.11 ± 1.44
Petroleum ether	43.65 ± 2.63	63.09 ± 3.65
Methanolic	48.97 ± 7.97	141.25 ± 1.16
Dichloromethane	53.70 ± 7.94	125.89 ± 7.86
Aqueous	69.18 ± 3.10	177.82 ± 4.07
Depleted aqueous	87.09 ± 2.39	158.48 ± 1.23

## Data Availability

All data are available in the laboratory of Physiology, Genetic and Ethnopharmacology, Faculty of Sciences, Mohammed the First University, PB 717, 60000, Oujda, Morocco, and Biology Unit and Medical Research CNESTEN, Rabat 10001, Morocco.
